# Effect of resting pressure on the estimate of cerebrospinal fluid outflow conductance

**DOI:** 10.1186/2045-8118-8-15

**Published:** 2011-03-07

**Authors:** Kennet Andersson, Nina Sundström, Jan Malm, Anders Eklund

**Affiliations:** 1 Department of Radiation Sciences, Umeå University, Umeå, Sweden; 2 Department of Clinical Neuroscience, Umeå University, Umeå, Sweden; 3 Centre of Biomedical Engineering and Physics, Umeå University, Umeå, Sweden

## Abstract

**Background:**

A lumbar infusion test is commonly used as a predictive test for patients with normal pressure hydrocephalus and for evaluation of cerebrospinal fluid (CSF) shunt function. Different infusion protocols can be used to estimate the outflow conductance (*C*_out_) or its reciprocal the outflow resistance (*R*_out_), with or without using the baseline resting pressure, *P*_r_. Both from a basic physiological research and a clinical perspective, it is important to understand the limitations of the model on which infusion tests are based. By estimating *C*_out_ using two different analyses, with or without *P*_r_, the limitations could be explored. The aim of this study was to compare the *C*_out_ estimates, and investigate what effect *P*_r_had on the results.

**Methods:**

Sixty-three patients that underwent a constant pressure infusion protocol as part of their preoperative evaluation for normal pressure hydrocephalus, were included (age 70.3 ± 10.8 years (mean ± SD)). The analysis was performed without (*C*_excl Pr_) and with (*C*_incl Pr_) P_r_. The estimates were compared using Bland-Altman plots and paired sample *t*-tests (*p *< 0.05 considered significant).

**Results:**

Mean *C*_out_ for the 63 patients was: *C*_excl Pr _= 7.0 ± 4.0 (mean ± SD) μl/(s kPa) and *C*_incl Pr_ = 9.1 ± 4.3 μl/(s kPa) and *R*_out_ was 19.0 ± 9.2 and 17.7 ± 11.3 mmHg/ml/min, respectively. There was a positive correlation between methods (r = 0.79, n = 63, *p *< 0.01). The difference, Δ*C*_out_= -2.1 ± 2.7 μl/(s kPa) between methods was significant (*p *< 0.01) and Δ*R*_out _was 1.2 ± 8.8 mmHg/ml/min). The Bland-Altman plot visualized that the variation around the mean difference was similar all through the range of measured values and there was no correlation between Δ*C*_out _and *C*_out_.

**Conclusions:**

The difference between *C*_out _estimates, obtained from analyses with or without *P*_r_, needs to be taken into consideration when comparing results from studies using different infusion test protocols. The study suggests variation in CSF formation rate, variation in venous pressure or a pressure dependent *C*_out _as possible causes for the deviation from the CSF absorption model seen in some patients.

## Background

Patients with normal pressure hydrocephalus (NPH) are treated with and often improved by a cerebrospinal fluid (CSF) shunt that changes the dynamics of the CSF system [1-4]. In order to assist in the selection of patients likely to benefit from shunt surgery, predictive tests are performed [5]. One such test is the infusion test. It measures changes in intracranial pressure due to infusion or withdrawal of Ringer solution. For clinical interpretation, the relation between pressure and flow obtained during an infusion test must be quantified into accessible parameters, i.e. a model of the CSF system is needed.

In the early seventies, Davson presented a model of the CSF absorption [6,7]. This has since been widely accepted and is used as one part of the model describing the dynamics of the CSF system:

(1) $$I_{\text{a}}=\left(P_{\text{ic}}-P_{\text{d}}\right)C_{\text{out}}=\frac{P_{\text{ic}}-P_{\text{d}}}{R_{\text{out}}}$$

Thus, it states that the rate of absorption (*I*_a_) is proportional to the difference between the pressure in the subarachnoid space (*P*_ic_) and venous pressure in dural sinus (*P*_d_). The proportionality coefficient is the outflow conductance (*C*_out_), or its reciprocal, the outflow resistance (*R*_out_). *C*_out _describes the ease of flow across the CSF outflow pathways. In addition to being used as a prognostic parameter for selecting patients responding to CSF shunt surgery, infusion measurement of *C*_out _is also used for evaluation of CSF shunt function [5,8-11].

To use equation (1) in the analysis of an infusion test, *P*_d_, which is difficult to measure, can be replaced by the measureable baseline resting pressure *P*_r_. To replace *P*_d _with *P*_r_, three assumptions are needed, that *C*_out _is a physical property independent of pressure and that the variations in *P*_d _and CSF formation rate, *I*_f_, during the infusion test are sufficiently small for *P*_d _and *I*_f _to be approximated as constants. If the variations in *P*_d_, *I*_f _and *C*_out _are negligible, the relationship between steady state pressure and net infusion flow should be linear. Since a model is never better than the validity of its assumptions, it is important to understand the effects on estimated *C*_out _caused by unfulfilled assumptions.

**Figure 1:**
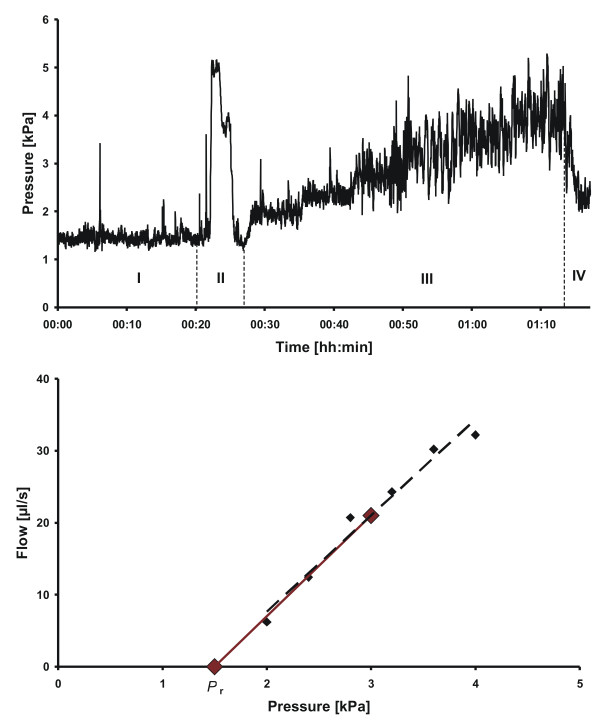
**Upper plot of pressure against time for one experiment: the infusion investigation starts with measurement of *P*_r _(I), CSF sampling with patient in sitting position (II), CPI protocol with six elevated pressure levels back in supine position (III) and a relaxation phase (IV)**. Lower plot of estimated flow against pressure: Results from the patient measurement illustrating the two analysis methods graphically. Lower red dot is measured *P_r_*, upper red dot is mean of the six black dots which are measured flow and pressure from the elevated pressure levels. The dotted black regression line of the six elevated levels illustrate method 1, the red line, connecting *P*_r _and the mean of the elevated levels, illustrate method 2. The slopes of the lines give the *C*_out _estimates respectively.

There are different infusion protocols, one such is the constant pressure infusion (CPI) protocol. It measures *P*_r _and six elevated pressure levels together with corresponding net flow [12]. With this particular protocol, as opposed to the commonly used constant infusion protocol [13], a more detailed pressure/flow relationship can be plotted. As mentioned, data is expected to form a straight line throughout the pressure range with a trajectory through *P*_r _and with the slope corresponding to *C*_out _(Figure 1). However, from clinical experience it is suspected that the regression line does not always pass through *P*_r_.

To understand the limitations of the current model used in infusion tests is important, both for basic physiological research and for clinical purposes. These limitations could be explored by comparing *C*_out _estimates calculated using two different analyses, one that included *P*_r _and one that did not. The aim of this study was to investigate how the use of baseline resting pressure influences the estimate of *C*_out_.

## Methods

### Patient population

The study population consisted of patients that underwent preoperative evaluation for NPH. All patients had an MRI that revealed ventriculomegaly (Evans ratio > 0.3) and they were without any visual obstruction to CSF flow. Sixty-three patients (age 70.3 ± 10.8 years (mean ± SD), 18 women) underwent a CPI protocol. The study has been reviewed by the Regional Ethical Review Board in Umeå who concluded that there were no ethical problems with the project.

### Infusion apparatus and investigation

The highly standardized infusion apparatus has been thoroughly described previously [12]. Two needles were inserted in the spinal canal while the patient was in the sitting position, one needle was used for pressure measurement and the other for infusion or withdrawal of Ringer solution. The patient was placed in the supine position and the zero-pressure reference level was placed at the level of the auditory meatus. The investigation is illustrated in Figure 1. First, *P*_ic _was measured during 15-20 minutes of rest, and *P*_r _was calculated as the mean *P*_ic _over the last five minutes. To ensure a stable measurement of *P*_r_, the patient was lying comfortably in supine position during the investigation, the importance of minimizing leakage during lumbar puncture was accentuated to the physician and the routine sample of CSF was taken after the measurement of *P*_r_. Following the *P*_r _measurement, the CPI protocol was initiated. *P*_ic _was increased to six, consecutive, predetermined pressure levels lasting seven minutes each (Figure 1) followed by a spontaneous relaxation phase.

### Estimation of C_out_

**Figure 2:**
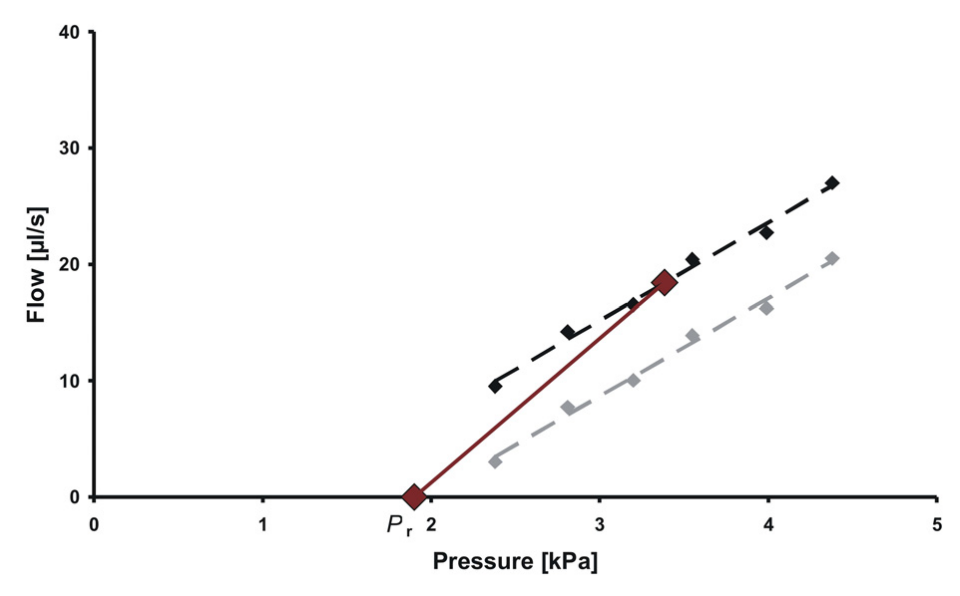
**Results illustrating a patient with large difference between methods**. Lower red dot is measured *P*_r _and upper large red dot is mean of the six black dots. The dotted black line is the estimate of *C*_excl Pr_, the red line is *C*_incl Pr_. Lower grey points with regression line illustrate a possible result without needed extra net flow. This typical pattern of extra net flow was visually observed for approximately one third of the patients.

The CSF absorption is estimated from Davson's equation (1). The two estimation methods used in this study are described below and illustrated in Figure 1 and Figure 2. They are derived from the model of CSF absorption and a CSF system in steady state. The assumption of conservation of fluid in the CSF system can be stated as

(2) $$I_{\text{f}}+I_{\text{ext}}=I_{\text{a}}+I_{\text{s}}$$

where *I*_f _is the formation rate, *I*_ext _is the infusion rate of a possible external infusion, *I*_a _is the rate of absorption and *I*_s _is the rate of change of fluid stored in the system. The normal unperturbed baseline resting pressure, *P*_r_, (*I*_s _and *I*_ext _equal to zero) of the patient is defined as

(3) $$P_{\text{r}}=P_{\text{d}}+\frac{I_{\text{f}}}{C_{\text{out}}}$$

When in steady state during an infusion test, *I*_a _= *I*_ext _+ *I*_f_, see equation (2). Combining this with equations (1) and (3), the relation between *I*_ext _and *P*_ic _is

(4) $$I_{\text{ext}}=C_{\text{out}}\left(P_{\text{ic}}-P_{\text{r}}\right)$$

### Method 1, analysis without P_r_

On each of the six elevated pressure levels, mean *P*_ic _as well as the net inflow (*I*_ext_) needed to maintain a constant *P*_ic _was measured. The relation between *I*_ext _and *P*_ic _was

(5) $$I_{\text{ext}}=C_{\text{excl}\mathrm{Pr}}P_{\text{ic}}+\text{constant}$$

*C*_excl Pr _was estimated as the slope of the linear regression between *I*_ext _and *P*_ic _using the six elevated pressure levels [12,14] (Figure 1).

### Method 2, analysis with P_r_

Pressure and flow from all six elevated levels, but without using the *P*_r_, were averaged into one pressure and flow point (${\bar{P}}_{\text{ic}}$ and $I_{\text{ext}}$respectively). *C*_incl Pr _was calculated as

(6) $$C_{\text{incl}\mathrm{Pr}}=\frac{{\bar{I}}_{\text{ext}}}{{\bar{P}}_{\text{ic}}-P_{\text{r}}}$$

i.e. a line was drawn between *P*_r _and ${\bar{P}}_{\text{ic}}$ and the slope corresponded to *C*_incl Pr _(Figure 1). The classic Katzman method of estimating *C*_out _during a constant infusion is achieved by dividing the mean flow with the difference between resting pressure and a pressure plateau [13]. The method for *C*_incl Pr _simulates that approach and uses the same formula.

### Statistics

Pearson's correlation coefficient was used for correlation analysis. The two estimates of *C*_out _were compared using Bland-Altman plots and paired sample *t*-tests, *p *< 0.05 was considered significant.

## Results

A typical infusion investigation is shown in Figure 1 with corresponding *C*_out _from the two methods. The mean outflow conductance for the 63 patients was *C*_excl Pr _= 7.0 ± 4.0 (mean ± SD) μl/(s kPa) (*R*_excl Pr _= 19.0 ± 9.2 mmHg/ml/min) and *C*_incl Pr _= 9.1 ± 4.3 μl/(s kPa) (*R*_incl Pr _= 17.7 ± 11.3 mmHg/ml/min) respectively. There was a positive correlation between the two methods (r = 0.79, n = 63, *p *< 0.01). The paired difference between estimation methods (Δ*C*_out _= *C*_excl Pr _- *C*_incl Pr_) was significant, Δ*C*_out _= -2.1 ± 2.7 μl/(s kPa), n = 63, *p *< 0.01 (Δ*R*_out _= 1.2 ± 8.8 mmHg/ml/min). The SD of Δ*C*_out _was 13% of the measurement range. Figure 2 illustrates a case where the difference between methods was large, Δ*C*_out _= 4.1 μl/(s kPa), is shown. Two phases were identified: 1. a net flow needed to raise the pressure from *P*_r _to the first level, 2. a pattern following a straight line from the first level to the sixth level.

**Figure 3:**
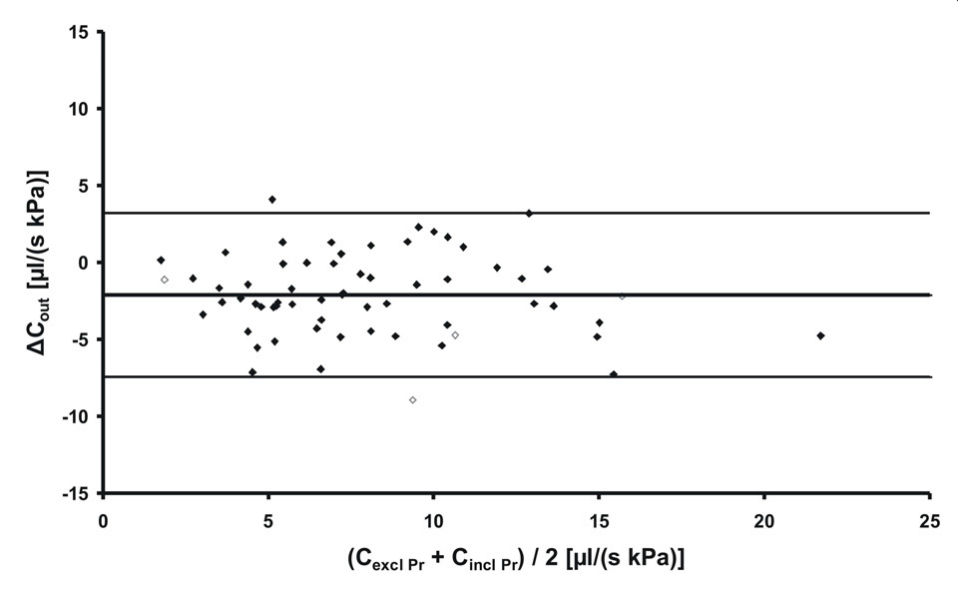
**A Bland-Altman plot of the two analysis methods for *C*_out _showing the difference Δ*C*_out, _vs. the average of the two methods for all subjects**. The lines are calculated as mean ± 1.96 SD. The open diamonds represent subjects with marked B-waves during *P*_r _measurement.

**Figure 4:**
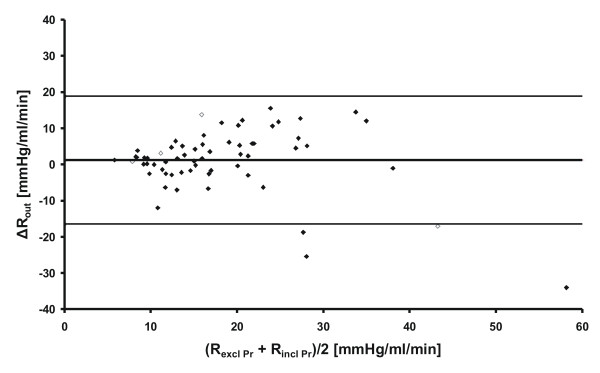
**A Bland-Altman plot of the two analysis methods for *R*_out _showing the difference Δ*R*_out _vs. the average of the two methods**. The lines are calculated mean ± 1.96 SD. The open diamonds represent subjects with marked B-waves during *P*_r _measurement.

The Bland-Altman plot in Figure 3 shows Δ*C*_out _plotted against the mean of the two analysis methods. The variation around the mean difference in *C*_out _was similar all through the range of measured pressures and there was no correlation between Δ*C*_out _and *C*_out_. A corresponding plot for *R*_out _is given in Figure 4.

## Discussion

This study investigated two analysis methods for estimating *C*_out_, with or without *P*_r_. The significant difference between the two methods (Figure 3) should be considered when comparing *C*_out _in studies using different methods and when setting threshold values for shunting. The correlation between methods was in the same range as between *C*_excl Pr _and *C*_out _from a previous study [15]. It should be noted that the difference between the two methods was small and similar to what has been found for repeated infusion protocols [12,15,16], therefore one has to be careful with regard to any clinical implications. Most analysis methods for infusion tests are based on the model and basic assumptions described in this paper, and current development of new analysis methods for pressure-controlled infusion will, as opposed to the CPI method used today, rely on *P*_r _[17]. It is therefore important to investigate the limitations of these assumptions and the effects they have on calculated *C*_out_.

The difference that was found depending on whether or not *P*_r _was used in the estimation of *C*_out_, (Figure 3), could be explained by several underlying causes. The infusion test analysis based on equation (1) assumes that *P*_d _and *I*_f _[18] are constant, but if they varied during the investigation, both *P*_r _and the estimation of *C*_out _would be affected. A potential explanation could be that the infusion of Ringer solution caused a physiological response with a reduction in *P*_d _and/or *I*_f _which would result in an increase of needed inflow as observed in this study (Figure 2), giving rise to the systematic difference in estimated *C*_out _depending on whether or not *P*_r _was used. Another assumption was that *C*_out _is constant and pressure independent. This assumption has been based on visual inspection or correlation coefficients of the pressure/flow relationship [19-24]. Specifically, a linear relationship was shown for a pressure interval of 0.7-1.6 kPa above *P*_r _[25], but that study focused on the use of *C*_excl Pr _and did not analyse the relationship down to *P*_r_. Other studies have proposed a nonlinear relationship between pressure and flow [26-28]. These studies suggested a continuously pressure dependent *C*_out _while in the present study, the results suggest that for certain patients (Figure 2), there was a higher *C*_out _in the vicinity of *P*_r _followed by a pressure independent *C*_out_. This could be explained by an active CSF outflow transport that starts when the system is perturbed by infusion, but with an absorption rate that is independent of further increases in pressure. This would indicate that the CSF outflow in the vicinity of *P*_r _in some cases may differ from the Davson equation.

It was not possible to deduce from this study which of *I*_f_, *P*_d _and a pressure independent *C*_out _was the major contributor to the systematic difference in results. The authors believe that the Davson equation is valid and that the deviation came from variations in *P*_d _and/or *I*_f _during the infusion. Monitoring of variation in central venous pressure during infusion tests could be a possible way forward. In addition to the systematic difference between methods, there was also a variation around the mean. This variation was probably mainly caused by the vascular effects on the CSF system (Figure 3). Vasomotion can cause large volume variations on the arterial side which in turn induce large pressure variations, e.g. B-waves [29]. The relatively small flows involved during an infusion test in comparison with these effects, will make the estimation of *C*_out _challenging. The steady-state analysis approach assumes that the dynamics of the system will be sufficiently suppressed by averaging over the 7 minutes of measurement time. However, the system dynamics for many patients can include components with potential to violate this assumption, e.g. B-waves or plateau waves, that can cause a reduction in accuracy of the estimated pressures and flows for the elevated levels [29,30]. These comparatively large physiological variations will also influence measured *P*_r_. Visual inspection of the *P*_r _measurements showed that four patients had marked B-waves. One of which was the subject with the highest difference between methods while the other three were method independent (Figure 3). Furthermore, pressure that had not stabilised enough during its 15-20 min baseline measurement, would also affect *P*_r_. This could be caused by apprehension of the patient. Another possibility was a slow formation rate unable to compensate for the loss of CSF during lumbar puncture. To avoid this, a routine was followed in order to obtain as reliable estimates as possible (see Methods section). Results of repeated measurements in the same patient with consecutive CPI and constant infusion protocols suggest that the vascular effects limit the expected precision for measurements with current infusion tests to approximately 2 μl/(s kPa) (SD) [12,15,16]. We interpret this as an inherent characteristic of the vascular and CSF system that limits the expected repeatability independently of which infusion method that is used.

Since *C*_incl Pr _uses an average value it will be less sensitive to physiological variations at the lowest or highest pressure levels. On the other hand it is dependent on *P*_r_, and an error in this parameter will have a major impact on the estimated *C*_out_, equation (6). Thus, the accuracy of estimated *P*_r _becomes essential. Furthermore, if results are compared with results from the constant infusion protocol with either static analysis according to Katzman [13] or dynamic analysis [31], *C*_incl Pr _should be used. Until future clinical studies have investigated the pressure/flow relationship in the vicinity of *P*_r _in more detail and its pathophysiological importance have been established, both methods are still relevant. An erroneous flow measurement could produce the shift upwards in flow (Figure 2). However, careful calibration and testing of the equipment on experimental set-up was performed [12,17], and these types of errors have not been observed.

## Conclusions

Using *P*_r _for estimating *C*_out _produced a higher estimated *C*_out_. Possible causes for a deviation from the model of CSF absorption in some patients were a variation in formation rate or venous pressure or a pressure dependent *C*_out_. The observed difference needs to be taken into consideration when setting threshold values for shunting and when comparing results from studies using different infusion test protocols.

## List of abbreviations

NPH: Normal pressure hydrocephalus; CSF: Cerebrospinal fluid; *P*_ic_: Intracranial pressure; *P*_r_: Resting pressure; *P*_d_: Venous pressure in dural sinus; *I*_a_: Absorption rate of CSF; *I*_f_: Formation rate of CSF; *I*_ext_: Rate of external infusion; *I*_s_: CSF stored in system; ${\bar{P}}_{\text{ic}}$: Average pressure; ${\bar{I}}_{\text{ic}}$; Average flow; *C*_excl Pr_: Outflow conductance estimated by method 1, without *P*_r_; *C*_incl Pr_: Outflow conductance estimated by method 2, with *P*_r_; Δ*C*_out_: Difference in conductance between methods

## Competing interests

Drs. Sundström, Malm, and Eklund have a patent interest in the in-house-developed infusion apparatus used in the study. Likvor AB has acquired the patent rights for commercialization.

## Authors' contributions

All authors participated in the conception and design of the study, collection of data, statistical analysis and critically revised the article, reviewed the final version of the manuscript and approved it for submission.
